# Endoscopic Transpapillary Stenting for Malignant Hilar Biliary Stricture: Side-by-Side Placement versus Partial Stent-in-Stent Placement

**DOI:** 10.3390/jpm13050831

**Published:** 2023-05-14

**Authors:** Koji Takahashi, Hiroshi Ohyama, Yuichi Takiguchi, Motoyasu Kan, Mayu Ouchi, Hiroki Nagashima, Izumi Ohno, Naoya Kato

**Affiliations:** 1 Department of Gastroenterology, Graduate School of Medicine, Chiba University, Chiba 260-8670, Japan; 2 Department of Medical Oncology, Graduate School of Medicine, Chiba University, Chiba 260-8670, Japan

**Keywords:** endoscopic retrograde cholangiopancreatography, hilar malignant biliary stricture, partial stent-in-stent, side-by-side, stent

## Abstract

Background/Aims: Endoscopic uncovered metal stent (UMS) placement has been widely performed for unresectable hilar malignant biliary stricture (UHMBS). Two stenting methods are used for the two bile duct branches: side-by-side placement (SBS) and partial stent-in-stent placement (PSIS). However, it remains controversial whether SBS or PSIS is superior. This study aimed to compare SBS and PSIS in UHMBS cases with UMS placement in two branches of the IHD. Methods: This retrospective study included 89 cases of UHMBS treated with UMS placement through the SBS or PSIS technique using endoscopic retrograde cholangiopancreatography at our institution. Patients were divided into two groups, SBS (*n* = 64) and PSIS (*n* = 25), and compared. Results: Clinical success was achieved in 79.7% and 80.0% in the SBS and PSIS groups, respectively (*p* = 0.97). The adverse event rate was 20.3% and 12.0% in the SBS and PSIS groups, respectively (*p* = 0.36). The recurrent biliary obstruction (RBO) rate was 32.8% and 28.0% in the SBS and PSIS groups, respectively (*p* = 0.66). The median cumulative time to RBO was 224 and 178 days in the SBS and PSIS groups, respectively (*p* = 0.52). The median procedure time was 43 and 62 min in the SBS and PSIS groups, respectively, which was significantly longer in the PSIS group (*p* = 0.014). Conclusions: No significant differences were noted in the clinical success rate, adverse event rate, time to RBO, or overall survival between the SBS and PSIS groups, other than the significantly longer procedure time in the PSIS group.

## 1. Background/Aims

Hilar malignant biliary stricture (HMBS) is caused by cholangiocarcinoma, gallbladder carcinoma, hepatocellular carcinoma, metastatic liver tumors, or hilar lymph node metastases from various cancers. HMBSs induce high serum bilirubin concentrations owing to cholestasis, which is called obstructive jaundice. Obstructive jaundice affects the biliary tree, hepatic cells, and liver functions. Additionally, the loss of bile in the gut disrupts the intestinal mucosal barrier, which increases the absorption of endotoxins from the intestine [1]. The symptoms of obstructive jaundice include itching, loss of appetite, and weight loss. Effective biliary drainage is required to improve the quality of life of HMBS patients. Although percutaneous biliary drainage and surgical biliary drainage are also available, endoscopic biliary drainage is recommended because it is less invasive. Endoscopic biliary drainage includes transpapillary drainage using endoscopic retrograde cholangiopancreatography (ERCP) and transgastrointestinal drainage using endoscopic ultrasonography. Unless there is a special reason, such as duodenal obstruction or postoperative gastrointestinal reconstruction, transpapillary drainage using ERCP is the first choice. Transpapillary drainage by ERCP involves endoscopic biliary stenting and endoscopic nasobiliary drainage. In unresectable HMBS (UHMBS), endoscopic biliary stenting is usually performed. Two types of biliary stents are used for endoscopic biliary stenting: plastic stents and metal stents. Several studies have shown that metal stents outperform patients with unresectable UHMBS in terms of patency and cost efficiency [2,3]. Thus, endoscopic uncovered metal stent (UMS) placement has been widely performed for UHMBS. Previously, unilateral UMS placement was the mainstream treatment for biliary drainage in UHMBS [4]. However, biliary drainage of >50% of the liver volume was reported to be associated with effective drainage for UHMBS in several studies [5,6]. Furthermore, a recent randomized controlled trial revealed better stent patency with bilateral UMS placement than with unilateral UMS placement for UHMBS [7]. Therefore, bilateral UMS placement is typically performed for UHMBS.

**Figure 1:**
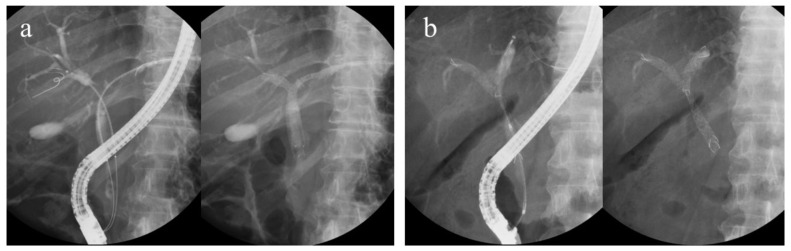
(**a**) Side-by-side placement and (**b**) partial stent-in-stent placement.

Regarding bilateral UMS placement using the ERCP, two stenting methods are used: side-by-side placement (SBS) and partial stent-in-stent placement (PSIS). SBS is a procedure in which two guidewires are placed in two branches of the intrahepatic bile duct (IHD), and then two stent deliveries are inserted and deployed sequentially. The routes of the two UMSs are independent of each other. Meanwhile, PSIS is a procedure in which the first stent is deployed, followed by another stent inserted through the mesh of the first stent and deployed (Figure 1). As a procedure, the PSIS is more complex.

Several reports have compared SBS and PSIS, but no conclusion has been drawn as to which is superior, and it remains controversial. Most previous reports indicate that there is no difference in performance between SBS and PSIS; however, they have been from high-volume centers, and their results might not be universal. This is because the majority of procedures may have been performed only by expert endoscopists. At our institution, there are many opportunities for trainees to perform endoscopic treatment under the supervision of experts; therefore, we decided to examine features of SBS and PSIS, including the endoscopist’s proficiency. This study aimed to compare SBS and PSIS in UHMBS cases with UMS placement in two branches of the IHD.

## 2. Materials and Methods

### 2.1. Study Design

This retrospective study was conducted at a single center. A total of 89 patients with UHMBS treated with UMS placement in two branches of the IHD using the ERCP technique between April 2006 and December 2022 were studied from the database of Chiba University Hospital. HMBS was diagnosed based on computed tomography (CT) and histopathological examination. In cases where histopathological malignant findings were unclear or histopathological examination was difficult, the diagnosis was made if a typical CT image could be confirmed. The resectability was judged comprehensively from the findings, including CT images and cholangiography. The exclusion criteria were as follows: (1) patients aged <20 years; (2) patients with percutaneous transhepatic biliary drainage continued even after UMS placement; (3) patients with Bismuth type I; (4) patients with biliary tract reconstruction; (5) patients with stent placements in three or more branches of the IHD in a single ERCP session; (5) patients with unclear background and treatment information; and (6) patients judged as inappropriate by the investigators. This study was approved by the ethics committee of our institution. Written informed consent for the procedure was obtained from all patients.

Participants were divided into two groups: the SBS group and the PSIS group. Baseline characteristics and clinical outcomes were obtained from the patients’ medical records and compared between the two groups. The baseline characteristics included age, sex, the proportion of direct UMS placement at the first ERCP session, pre-ERCP serum total bilirubin level, pre-ERCP serum albumin level, Bismuth type, extrahepatic bile duct diameter in the tumor-free area before stent placement, the causes of malignant biliary obstruction, the proportion of above the papilla stent placement, endoscopist proficiency, and the proportion of chemotherapy after UMS placement. The clinical outcomes included procedure time, clinical success rate, adverse event rate, recurrent biliary obstruction (RBO) rate, cumulative time to RBO (TRBO) in effective drainage cases, re-intervention rate, technical success rate of the first re-intervention, procedure time of the first re-intervention, and overall survival.

### 2.2. Definitions

Clinical success, RBO, and adverse events were defined according to the Tokyo criteria 2014 [8]. Clinical success was defined as a decrease in the serum total bilirubin level to <50% or <2.0 mg/dL within 14 days of stent placement, without additional biliary drainage. RBO was defined as a composite endpoint, including stent occlusion and migration. TRBO refers to the time from stent placement to RBO. Deaths without RBO were treated as censored at the time of death. Patients lost to follow-up without RBO were treated as censored at the time of the final follow-up. Stent occlusion was defined as the occurrence of jaundice with cholestasis and evidence of bile duct dilation on imaging or endoscopy. Procedure time was defined as the time interval between insertion of the endoscope into the mouth and its removal. The extrahepatic bile duct diameter in the tumor-free area was measured on cholangiography during ERCP. In re-intervention, technical success was defined as additional stent placement or balloon sweep for a UMS judged to be obstructed by CT images.

### 2.3. Techniques

Placement of the UMS was performed transpapillary using the ERCP technique. Prior to UMS placement, CT was performed, and the biliary drainage area was planned based on the liver volume in all cases. Prophylactic intravenous antibiotics were administered before the procedure. ERCP was performed using an oblique-viewing endoscope (JF-260V, TJF-260V, and TJF-Q290V; Olympus, Tokyo, Japan). In the absence of contraindications, carbon dioxide insufflation was used during ERCP. All patients underwent intravenous sedation. Sodium meglumine amidotrizoate was used as a contrast medium. An endoscope was inserted orally to reach the duodenal papilla, and an ERCP catheter (PR-104Q-1; Olympus, Tokyo, Japan. MTW; ABIS, Hyogo, Japan. SwingTip; Olympus, Tokyo, Japan) was inserted into the bile duct. After cholangiography and identifying the site of biliary stenosis with a contrast medium, guidewires (VisiGlide; Olympus, Tokyo, Japan. VisiGlide 2; Olympus, Tokyo, Japan. M-Through; ASAHI INTECC, Aichi, Japan. EndoSelector; Boston Scientific, Marlborough, MA, USA) were placed in two branches of the IHD. Subsequently, UMSs (Zilver 635 Biliary Stent; Cook Medical, Bloomington, IN, USA. X-Suit NIR; Olympus, Tokyo, Japan. WallFlex; Boston Scientific, Marlborough, MA, USA. Niti-S D-type; Century Medical, Tokyo, Japan. BileRush; Piolax, Yokohama, Japan. JOSTENT; Zeon Medical, Tokyo, Japan. Zeo Stent V; Zeon Medical, Tokyo, Japan. YABUSAME; Kaneka, Osaka, Japan.) were deployed in two branches of the IHD. For SBS, after the insertion of the guidewires into the two branches of the IHD, two stent deliveries were inserted into the IHDs and sequentially deployed. For PSIS, the first stent was inserted into one IHD after the insertion of guidewires into the two IHDs. Subsequently, after deploying the first stent, the guidewire was passed through the mesh of the first stent into another IHD. The second stent was deployed through the mesh of the first stent. All procedures were performed by experts on ERCP (≥10 years of ERCP experience) or by trainees (<10 years of ERCP experience) under the supervision of experts. If the operator changed during the procedure, the physician who had operated the endoscope for a long time was defined as the operator of the procedure.

### 2.4. Statistical Analysis

Data were presented as median with interquartile range or number with a percentage. For the univariate analysis, the Mann–Whitney U test was used to compare continuous variables, whereas Pearson’s Chi-squared test was used to compare categorical variables. Cumulative TRBO and overall survival were estimated using Kaplan–Meier analysis, and Kaplan–Meier curves were compared using the log-rank test. Statistical significance was set at *p* < 0.05. All statistical analyses were performed using Bell Curve for Excel (Social Survey Research Information Co., Ltd., Tokyo, Japan).

## 3. Results

The age of the study participants ranged from 36 to 90 years, with a median of 71 (64–79) years. Of the 89 patients, 64 underwent SBS, and 25 underwent PSIS. All patients underwent endoscopic sphincterotomy before UMS placement.

Patient characteristics of the two groups are presented in Table 1. The median age was 71 (66–79) and 71 (63–79) years in the SBS and PSIS groups, respectively (*p* = 0.84). The proportion of men in the SBS and PSIS groups was 67.2% and 64.0%, respectively (*p* = 0.78). The proportions of direct UMS placement at the first ERCP session were 15.6% and 28.0% in the SBS and PSIS groups, respectively (*p* = 0.18). The median pre-ERCP serum total bilirubin level was 4.8 (1.4–8.4) and 8.1 (2.1–11.6) mg/dL in the SBS and PSIS groups, respectively (*p* = 0.12). The median pre-ERCP serum albumin level was 2.7 (2.4–3.2) and 3.0 (2.5–3.7) mg/dL in the SBS and PSIS groups, respectively (*p* = 0.17). Regarding the Bismuth type, the proportion of type II was 15.6% and 8.0% in the SBS and PSIS groups, respectively (*p* = 0.34). The proportions of type IIIa were 20.3% and 44.0% in the SBS and PSIS groups, respectively, and significantly higher in the PSIS group (*p* = 0.024). The proportions of type IIIb were 15.6% and 20.0% in the SBS and PSIS groups, respectively (*p* = 0.62). The proportions of type IV were 48.4% and 28.0% in the SBS and PSIS groups, respectively (*p* = 0.080). The median extrahepatic bile duct diameter in the tumor-free area was 8.7 (7.2–9.9) and 6.6 (6.1–7.2) mm in the SBS and PSIS groups, respectively, significantly larger in the SBS group (*p* < 0.01). Regarding causes of malignant biliary obstruction, the proportion of hilar cholangiocarcinoma was 48.4% and 48.0% in the SBS and PSIS groups, respectively (*p* = 0.97). The proportion of gallbladder cancer in the SBS and PSIS groups was 17.2% and 28.0%, respectively (*p* = 0.25). The proportions of intrahepatic cholangiocarcinoma in the SBS and PSIS groups were 6.3% and 4.0%, respectively (*p* = 0.68). The proportions of hepatocellular carcinoma in the SBS and PSIS groups were 10.9% and 12.0%, respectively (*p* = 0.89). The proportion of metastatic liver tumors in the SBS and PSIS groups was 10.9% and 8.0%, respectively (*p* = 0.68). The proportions of hilar lymph node metastases in the SBS and PSIS groups were 6.3% and 0%, respectively (*p* = 0.20). The proportion of patients above-the-papilla stent placement in the SBS and PSIS groups was 90.6% and 88.0%, respectively (*p* = 0.71). The proportions of expert performance in the SBS and PSIS groups were 20.3% and 48.0%, respectively, and significantly higher in the PSIS group (*p* < 0.01). The proportions of chemotherapy after UMS placement were 32.8% and 36.0% in the SBS and PSIS groups, respectively (*p* = 0.78). No significant differences were noted in terms of age, sex, the proportion of direct UMS placement at the first ERCP session, pre-ERCP serum total bilirubin level, pre-ERCP serum albumin level, the proportion of the causes of malignant biliary obstruction, the proportion of above the papilla stent placement, or the proportion of chemotherapy after UMS placement between the two groups. 

Clinical outcomes in the SBS and PSIS groups are shown in Table 2. The median procedure time was 43 (28–53) and 62 (40–74) min in the SBS and PSIS groups, respectively, and significantly longer in the PSIS group than in the SBS group (*p* = 0.014). Clinical success was achieved in 79.7% and 80.0% in the SBS and PSIS groups, respectively (*p* = 0.97). The adverse event rate was 20.3% and 12.0% in the SBS and PSIS groups, respectively (*p* = 0.36). Regarding adverse events, the proportion of cholangitis in the SBS and PSIS groups was 7.8% and 4.0%, respectively (*p* = 0.52). The proportions of pancreatitis in the SBS and PSIS groups were 6.3% and 4.0%, respectively (*p* = 0.68). The proportions of liver abscesses in the SBS and PSIS groups were 3.1% and 0%, respectively (*p* = 0.37). The proportions of pneumonia in the SBS and PSIS groups were 1.6% and 4.0%, respectively (*p* = 0.49). The proportions of heart failure in the SBS and PSIS groups were 1.6% and 0%, respectively (*p* = 0.53).

**Figure 2:**
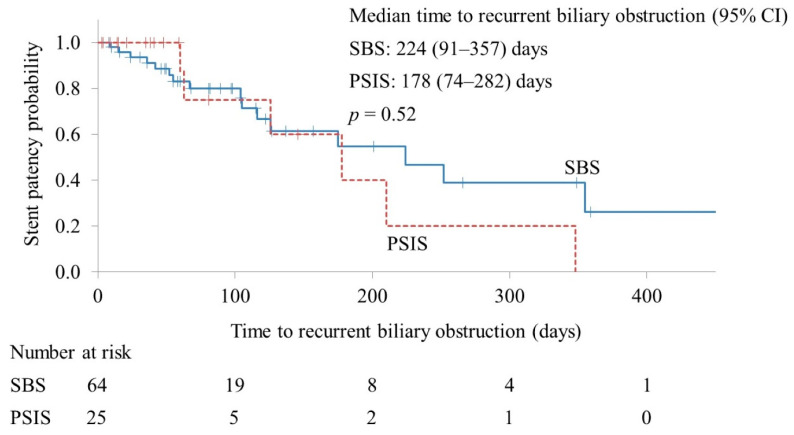
Kaplan–Meier curves of the time to recurrent biliary obstruction. CI, confidence interval; SBS, side-by-side placement; PSIS, partial stent-in-stent placement.

The RBO rates in the SBS and PSIS groups were 32.8% and 28.0%, respectively (*p* = 0.66). Stent occlusion was the only cause of RBO, and there were no cases of stent migration. The median cumulative TRBO in the effective drainage cases was 224 and 178 days in the SBS and PSIS groups, respectively (*p* = 0.52) (Figure 2).

**Figure 3:**
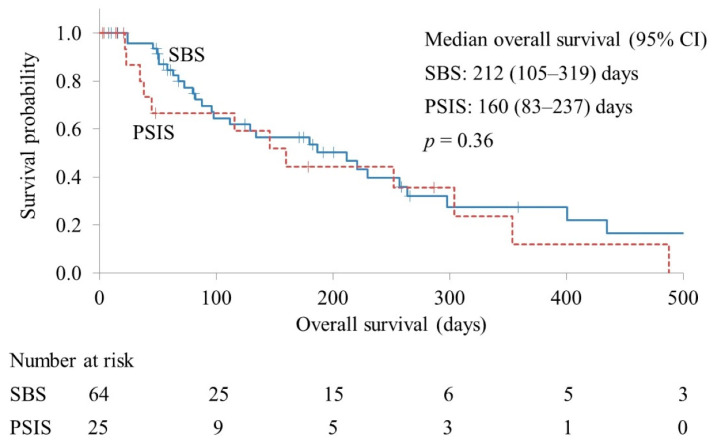
Kaplan–Meier curves of overall survival. CI, confidence interval; SBS, side-by-side placement; PSIS, partial stent-in-stent placement.

The re-intervention rates in the SBS and PSIS groups were 31.2% and 28.0%, respectively (*p* = 0.76). The technical success rates of the first re-intervention were 90.0% and 100% in the SBS and PSIS groups, respectively (*p* = 0.38). The median procedure time of the first re-intervention was 39 and 37 min in the SBS and PSIS groups, respectively (*p* = 0.76). The median overall survival was 212 and 160 days in the SBS and PSIS groups, respectively (*p* = 0.36) (Figure 3).

## 4. Discussion

In this study, we compared SBS and PSIS in UHMBS cases treated with UMS placement in two branches of the IHD and found that there was no significant difference in the clinical success rate, adverse event rate, RBO rate, cumulative TRBO in effective drainage cases, re-intervention rate, technical success rate of the first re-intervention, procedure time of the first re-intervention, or overall survival between the SBS and PSIS groups other than that the procedure time was significantly longer in the PSIS group, despite the higher rate of expert enforcement in PSIS compared to SBS. Adverse events included cholangitis, pancreatitis, liver abscess, pneumonia, and heart failure, none of which occurred disproportionately in either SBS or PSIS.

In this study, the rate of expert enforcement was significantly higher in PSIS than in SBS, probably because the procedure was more complex in PSIS, so there were more opportunities for experts to perform it. The reason for the predominance of Bismuth IIIa in PSIS is unclear. There is no set standard for the proper use of SBS and PSIS, and it depends on the policy of each institution. In PSIS, there is little concern regarding bile duct overdilation. Meanwhile, in SBS, the bile ducts are overextended due to the parallel insertion of the two stents, raising concerns such as portal vein obstruction. However, there were no such adverse events in this study. 

Unilateral stenting was reported to be sufficient for effective biliary drainage in patients with UHMBS in two studies published in 2003 [4,9]. However, several reports have subsequently reported that drainage of >50% of the variable liver volume is an important drainage predictor and signals effective palliation in patients with UHMBS [5,6]. In recent years, several studies have reported the efficacy of multiple stenting for UHMBS [7,10,11,12,13,14]. To date, there are only five reports comparing SBS and PSIS, except for a meta-analysis. In 2012, Naitoh reported that deploying SBS resulted in a higher frequency of early and late adverse events than deploying PSIS placement. However, cumulative stent patency was prolonged in the SBS group in their single-center retrospective study (28 patients with SBS and 24 patients with PSIS) [15]. In 2012, Kim reported that adverse events, stent patency, and survival were not significantly different between the SBS and PSIS groups in a single-center retrospective study (19 patients with SBS and 22 patients with PSIS) [16]. In 2019, Lee reported that the efficacy of bilateral SIS and SBS placement was similar in terms of total adverse event rate, technical and clinical success, and stent patency in a randomized controlled trial (35 patients with SBS and 34 patients with PSIS) [17]. In 2020, Ishigaki reported that the clinical outcomes of the SBS and PSIS methods were comparable in patients with unresectable hilar malignant biliary obstruction in their multicenter retrospective study (24 patients with SBS and 40 patients with PSIS) [18]. In 2022, Iwai reported that the technical success rate, procedure duration, clinical success rate, adverse event rate, TRBO, and RBO rate were not significantly different between the SIS and SBS groups. However, endoscopic re-stenting after RBO was significantly more successful in the SBS group than in the SIS group in a single-center retrospective study (30 patients with SBS and 75 patients with PSIS) [19]. Few studies have examined the proficiency of endoscopists. In a retrospective report by Ishigaki, similar to this study, the proportion of experts performing PSIS was significantly higher than that performing SBS; however, in the study, there was no significant difference in the procedure time between SBS and PSIS [18].

In our study, procedure times were significantly longer in the PSIS group, despite the high percentage of procedures performed by expert endoscopists in the PSIS group. This is considered to be due to the fact that the procedure is more complex in PSIS than in SBS. In this study, there was a 19 min difference in the median procedure time between the SBS and PSIS groups. Few studies have investigated the relationship between the incidence of adverse events and procedure time in ERCP. In 2014, Mehta compared 177 ERCP procedures with <45 min to 118 ERCP procedures with >45 min and found no significant difference in the occurrence rates of adverse events [20]. Although not ERCP, in 2016, Kawanishi reported that a procedure time of 30 min or longer was a significant risk factor in the development of aspiration pneumonia after endoscopic hemostasis for upper gastrointestinal bleeding [21]. In 2022, Park reported that a procedure time of 80 min or longer in gastric endoscopic submucosal dissection is a risk factor for the onset of aspiration pneumonia after the procedure [22]. Although there are no clear data for ERCP, there is a concern that the risk of aspiration pneumonia increases with prolonged procedure time in ERCP. In particular, in patients at a high risk of aspiration pneumonia, such as the elderly or those with poor performance status, it may be preferable to choose SBS over PSIS. However, with a median difference of 19 min, it is difficult to conclude that PSIS is inferior to SBS. Therefore, either SBS or PSIS placement can be selected according to each endoscopist’s expertise or preference.

Our study has some limitations. The first is its retrospective design. The method of stent placement was not randomized. There is a difference in the background between the SBS group and the PSIS group. Although propensity score matching is useful to match the backgrounds of both groups, it was not performed in this study due to the small sample size. Second, various stents are used, and clinical outcomes may differ according to stent type. It would be ideal to compare SBS and PSIS using the same type of stent, but in practice, it is difficult to secure a sufficient sample size. Third, our study population, which comprised patients with different types of diseases, was heterogeneous. The time to stent occlusion may vary depending on the type of disease that causes UHMBS. To resolve this limitation, it is desirable to conduct the investigation for hilar cholangiocarcinoma only. However, it is still difficult to secure a sufficient sample size. Finally, the treatment of the second or third RBO has not been evaluated. Further analysis including long-term follow-up is necessary to mitigate this limitation.

## 5. Conclusions

PSIS has a significantly longer procedure time, despite the higher rate of expert enforcement in PSIS than in SBS. Moreover, the procedure of choice may be selected based on the endoscopist’s experience and preference.

## Figures and Tables

**Table 1:** Patient characteristics of the two groups.

	SBS Group	PSIS Group	*p*-Value
	*n* = 64	*n* = 25	
Age, year, median (IQR)	71 (66–79)	71 (63–79)	0.84
Sex, man, *n* (%)	43 (67.2%)	16 (64.0%)	0.78
Direct UMS placement at the first ERCP session, *n* (%)	10 (15.6%)	7 (28.0%)	0.18
Pre-ERCP serum total bilirubin level, mg/dL, median (IQR)	4.8 (1.4–8.4)	8.1 (2.1–11.6)	0.12
Pre-ERCP serum albumin level, mg/dL, median (IQR)	2.7 (2.4–3.2)	3.0 (2.5–3.7)	0.17
Bismuth type, *n* (%)			
Type II	10 (15.6%)	2 (8.0%)	0.34
Type IIIa	13 (20.3%)	11 (44.0%)	0.024
Type IIIb	10 (15.6%)	5 (20.0%)	0.62
Type IV	31 (48.4%)	7 (28.0%)	0.080
Extrahepatic bile duct diameter in the tumor-free area before stent placement, mm, median (IQR)	8.7 (7.2–9.9)	6.6 (6.1–7.2)	<0.01
Causes of malignant biliary obstruction, *n* (%)			
Hilar cholangiocarcinoma	31 (48.4%)	12 (48.0%)	0.97
Gallbladder cancer	11 (17.2%)	7 (28.0%)	0.25
Intrahepatic cholangiocarcinoma	4 (6.3%)	1 (4.0%)	0.68
Hepatocellular carcinoma	7 (10.9%)	3 (12.0%)	0.89
Metastatic liver tumor	7 (10.9%)	2 (8.0%)	0.68
Hilar lymph nodes metastasis	4 (6.3%)	0	0.20
Above the papilla stent placement, *n* (%)	58 (90.6%)	22 (88.0%)	0.71
Operators, expert, *n* (%)	13 (20.3%)	12 (48.0%)	<0.01
Chemotherapy after UMS placement, *n* (%)	21 (32.8%)	9 (36.0%)	0.78

SBS, side-by-side placement; PSIS, partial stent-in-stent placement; IQR, interquartile range; UMS, uncovered metal stent; ERCP, endoscopic retrograde cholangiopancreatography.

**Table 2:** Clinical outcomes of the two groups.

	SBS Group	PSIS Group	*p*-Value
	*n* = 64	*n* = 25	
Procedure time, minutes, median (IQR)	43 (28–53)	62 (40–74)	0.014
Clinical success, *n* (%)	51 (79.7%)	20 (80.0%)	0.97
Adverse events, *n* (%)	13 (20.3%)	3 (12.0%)	0.36
Cholangitis	5 (7.8%)	1 (4.0%)	0.52
Pancreatitis	4 (6.3%)	1 (4.0%)	0.68
Liver abscess	2 (3.1%)	0	0.37
Pneumonia	1 (1.6%)	1 (4.0%)	0.49
Heart failure	1 (1.6%)	0	0.53
RBO, *n* (%)	21 (32.8%)	7 (28.0%)	0.66
Cumulative TRBO in effective drainage cases, days, median (95% CI)	224 (91–357)	178 (74–282)	0.52
Re-intervention, *n* (%)	20 (31.2%)	7 (28.0%)	0.76
Technical success rate of the first re-intervention, *n* (%)	18 (90.0%)	7 (100%)	0.38
Procedure time of first re-intervention, min, median (IQR)	39 (30–70)	37 (33–55)	0.76
Overall survival, days, median (95% CI)	212 (105–319)	160 (83–237)	0.36

SBS, side-by-side placement; PSIS, partial stent-in-stent placement; IQR, interquartile range; CI, confidence interval; RBO, recurrent biliary obstruction; TRBO, time to recurrent biliary obstruction.

## Data Availability

The datasets generated during and/or analyzed during the current study are not publicly available but are available from the corresponding author upon reasonable request.
